# Increasing urinary podocyte mRNA excretion and progressive podocyte loss in kidney contribute to the high risk of long-term renal disease caused by preterm birth

**DOI:** 10.1038/s41598-021-00130-y

**Published:** 2021-10-19

**Authors:** Fangrui Ding, Qi Gao, Xiuying Tian, Jiali Mo, Jun Zheng

**Affiliations:** 1 Department of Neonatology, Tianjin Central Hospital of Obstetrics and Gynecology, No. 156 Nan Kai San Ma Lu, Tianjin, 300000 People’s Republic of China; 2 Tianjin Key Laboratory of Human Development and Reproductive Regulation, Tianjin, 300000 People’s Republic of China; 3 Department of Neonatology, Nankai University Maternity Hospital, Tianjin, 300000 People’s Republic of China; 4 Graduate School, Tianjin Medical University, Tianjin, 300000 People’s Republic of China

**Keywords:** Cell biology, Developmental biology, Molecular biology, Nephrology

## Abstract

Podocyte abnormalities are common mechanism driving the progression of glomerular diseases, which account for most chronic kidney diseases (CKDs). However, the role of podocyte in the mechanism of high-risk long-term CKD caused by prematurity has not been well clarified. In present study, urine samples of 86 preterm infants and 32 full-term infants were collected, and podocyte-specific podocin mRNA levels in urine pellet were applied to indicate urinary podocyte mRNA excretion. In addition, in a preterm animal rat model, preterm rats were identified by delivery 2 days early. From the age of 3 weeks–12 months, urine samples were collected to examine podocyte mRNA excretion by measuring podocyte-specific podocin mRNA levels. Kidney samples at the age of 3 weeks, 2 months, and 12 months were collected from 8, 5 and 6 preterm rats and 9, 6 and 8 full-term rats, respectively, to examine podocyte density and podocyte area by measuring the podocyte specific nuclear marker WT-1 and the podocyte specific marker synaptopodin. As results, a more than threefold increase of urinary podocyte-specific podocin mRNA excretion rate was found in preterm infants compared with full-term infants. In addition, there was negative correlation between gestational age at birth and urinary podocin mRNA excretion. In preterm rats, a reduction in the total number of differentiated podocytes in glomeruli and an increased podocyte podocin mRNA excretion rate in urine were detected at the end of kidney differentiation. Moreover, long-term follow-up data in preterm rats showed there was an increased the risk of renal disease indicated by persistent podocyte mRNA loss, proteinuria, and enlarged glomeruli. In conclusion, increasing podocyte mRNA excretion in urine and podocyte loss in kidney led by prematurity drive the progression of long-term abnormal kidney function and could potentially explain the high risk of long-term CKD in preterm infants.

## Introduction

Chronic kidney disease (CKD) is a recognized public health issue and an important contributor to the global disease burden^1–3^. The identification of risk factors and the potential mechanism of these risk factors involved in CKD are key to preventing an increasing disease burden^4^. Prematurity is a risk factor for CKD and has been identified in the last three decades^5–7^. In 1999, Hoy et al. were the earliest researchers to report that low birth weight contributed to a higher albuminuria ratio and higher risk of renal disease in a large cohort although renal diseases were documented in very preterm infants in many case reports before this study^6,8^. Although gestational age information was not present in that study, most persons with low birth weight could be born prematurely (< 37 gestational weeks). Thus, that study suggests not only the role of low birth weight in the development of CKD but also the role of prematurity in the development of CKD^6^. Later, an increasing number of epidemiological studies reported a correlation between prematurity and CKD^9,10^. To date, the largest sample size of these kinds of studies was published by Crump et al. in 2019^7^. In this study, a national cohort of more than four million people was included to examine the relationship between preterm birth and long-term CKD risk. In this large national cohort study, researchers reported that preterm birth was a strong risk factor for the development of CKD from childhood to mid-adulthood. Compared with full-term birth, preterm birth had a twofold higher risk of CKD, and extremely preterm birth had a threefold higher risk of CKD^7^. The above studies verified that preterm birth is a risk factor for long-term CKD. However, the underlying mechanism of this process has not been well clarified.

Before published studies that indicated that prematurity is a risk factor for CKD, Brenner et al. have already proposed that a low nephron number could increase the risk of CKD by reducing kidney adaptive capacity^11^. This mechanism was the first to be proposed to explain this phenomenon and is currently believed to be the most likely mechanism for long-term kidney disease. Recent years, both human and animal studies have confirmed that premature birth has adverse effects on kidney development and nephron number^12–15^. In human autopsy studies, Rodríguez et al. described postnatal glomerulogenesis in extremely low birth weight preterm infants and concluded that the kidney continues to form after preterm birth, but glomerulogenesis is altered and fewer glomeruli are produced in preterm infants^12^. In Sutherland et al. study, the same finding that nephrogenesis is ongoing at the time of birth of preterm infants was detected and also preterm kidney may have fewer functional nephrons was indicated in that study^13^. Additionally, many animal models have confirmed premature birth and its effect on kidney development and nephron number^14,15^. In the Cary Stelloh et al. study, a preterm mouse model of pups delivered 1 or 2 days early by cesarean delivery was established, and after a few weeks of feeding, the glomeruli were counted. Fewer nephrons were present in preterm mice than in full-term animals^14^. Nevertheless, whether the reduction in the number of nephrons caused by preterm birth drives long-term kidney disease or increases the risk for kidney diseases has not been well examined.

Although CKD is caused by many different reasons, the major cause is glomerular diseases^16^. In the current mechanism of glomerular disease, podocytes play the most important role during the development and progression of glomerular disease^16–19^. Moreover, podocyte depletion has already been proven to be the cause of proteinuria and glomerulosclerosis, and persistent podocyte loss is the major risk factor driving glomerular disease progression^16,20–22^. Consequently, monitoring podocyte loss becomes important. Because it is difficult to detect podocyte number depletion in humans, urine podocyte products including urine pellet podocyte pellet mRNA were widely used in such cases^23–26^. Though urine pellet podocyte mRNA cannot be completely interpreted as podocyte depletion due to different cell size and synthetic activity (for example: quantitation of mRNA in one larger cell might be equivalent to mRNA in 2 smaller cells), previous studies have proven that excretion of urine pellet mRNA levels correlated strongly with rate of depletion of podocytes from glomeruli^17,25,26^. Larysa Wickman et al. reported that urine pellet podocyte mRNAs could be useful for monitoring risk for the progression of glomerular diseases^25^. Additionally, Yuji Sato et al. demonstrated that urine pellet mRNA can also be used to estimate the rate of podocyte depletion from glomeruli in humans^26^. In the present study, urine podocin mRNA extracted from urine pellet of preterm and full term infants and rats was used to indicate urinary podocyte mRNA depletion. In addition, examination of podocyte density and podocyte area in kidney biopsy samples has also been employed to examine the changes in podocyte loss in preterm rats.

## Methods

This study was approved by the Tianjin Central Hospital of Gynecology and Obstetrics Institutional Review Board (No. 2020KY049), Nankai University Animal Research Ethics Committee (No. 2018M0305). On human subjects, all study protocols adhered to the Declaration of Helsinki and written informed consent was received from all parents of participants prior to inclusion in the study. In animal studies, we confirmed that all the experimental protocols were approved by the Nankai University Animal Research Ethics Committee. In addition, all animal experiments were performed in accordance with the Regulations of Experimental Animal Administrations issued by the State Committee of Science and Technology of the Peoples Republic of China and ARRIVE guidelines about the use and care of experimental animals.

### Full-term and preterm infants

Infants born at Tianjin Central Hospital of Gynecology and Obstetrics from March 2019 to June 2019 were included in the present study. Infants with acute kidney injury, known kidney diseases or chromosomal or congenital anomalies were excluded. Infants were divided into two groups: a full-term group and a preterm group. After consent was obtained, 86 preterm infants and 32 full-term infants were enrolled. General information was obtained from the medical records.

### Human urine sample collection

Nephrogenesis in humans ends at approximately 36 weeks gestation^12,27,28^. For full-term infants, urine samples were collected at 4–7 days postnatal due to the creatinine influenced by maternal levels within 3 days postnatal. To obtain comparable age-matched urine samples, urine samples were collected at 37–40 weeks of corrected gestation in infants born preterm. During urine collection, each urine collection bag was used to collect urine at most 3 h and then removed from infants and stored at 4 °C. When the total volume of urine samples for each infant exceeded 30 ml, all urine collection bags were sent to the lab and pooled together for further processing. Urine volume of 24 h were acquired by weighing the diapers and adding the volume in collect bags.

### Establishment of a preterm rat model

Adult male and female Sprague–Dawley rats were purchased from Charles River Laboratories (Beijing). After 16 pairs of female and male rats were caged together at a 1:1 ratio, the vaginal plug was observed every 8 h, and the time of vaginal plug presence was marked as 0.5 days of gestational age. Due to the failure ratio of pregnant rats, 10 female rats with significant enlargement of the abdomen later days of vaginal plug presentation were included in further processes. At 20.5 days of gestational age, 5 pregnant female rats were randomly selected and then cesarean delivery was performed. A total of 43 pups were acquired from these 5 pregnant rats. During this process, the gravid uterus was removed, and the fetal membrane was artificially ruptured very quickly. Amniotic fluid was wiped dry with a cotton swab, and the umbilical cord was cut. All of these operations were performed carefully and gently. If the rat pup did not breathe spontaneously within 30–60 s, external chest compression was applied until spontaneous respiration was observed. We revived 95% of the pups, and they were alive until the endpoint. Finally, 40 pups were successfully resuscitated, and 6–8 preterm pups were distributed to female rats who delivered within 3 days for further suckling. Full-term control rats were obtained from vaginal spontaneous labor at 22.5 days of gestational age from another 5 pregnant rats. Finally, 45 full-term pups were acquired. The number of full-term pups was also limited to 6–8 for each mother. At the age of three weeks, male rats were chosen for further study in both the preterm and full term groups. Finally, 19 preterm male rats and 23 full-term male rats were included in the present study. At the age of 3 weeks, 2 months and 12 months, 8, 5 and 6 rats were sacrificed for the collection of kidney samples in the preterm rat group while 9, 6 and 8 rats were sacrificed for the collection of kidney samples in the full term rat group.

### Rat sample collection

All of the rats were weaned at 3 weeks. For urine samples, the rats were placed in metabolic cages for 24 h, and then urine samples were collected. Urine samples were collected every week within the age of 2 months and every month from 2 to 12 months. At 3 weeks, 2 months, and 12 months, 5–9 rats in each group were euthanized by CO_2_ exposure, and the kidneys were removed and fixed for further analysis.

### Both human and rat urine sample processing for RT-PCR

The methods used were as previously described^17,25,26^ and were detailed as follows. After urine collection, urine samples were centrifuged at 4 °C for 15 min at 4000 rpm. Two milliliters of supernatant were stored at − 20 °C for further creatinine and protein examination. Urine creatinine were determined by using a creatinine assay kit (BioAssay Systems Dict-500). Briefly, standard and urine samples were prepared firstly and then mixed with working reagent provided by assay kit. After reading the OD value at 510 nm, the creatinine concentration of the urine sample has been calculated. Then, the remaining supernatant was discarded. The pelleted urine was washed with DEPC-PBS to remove contaminating proteins and other soluble materials. After washing, the urine pellet in DEPC-PBS was centrifuged at 12,000 rpm for 5 min at 4 °C. Then, the supernatant was removed, and RLT buffer with β-mercaptoethanol was added based on the RNeasy RNeasy Mini Kit (Qiagen, 74106) protocol. The urine pellet/RLT was stored at − 80 °C or processed immediately for RNA extraction. RNA extraction was performed according to the manual instructions of the RNeasy Mini Kit (Qiagen, 74106). After extraction of RNA, quantitation of the absolute podocin mRNA abundance was performed using the 7500 Fast Real-Time PCR System (Applied Biosystems) using TaqMan Fast Universal PCR MasterMix, with sample cDNA in a final volume of 20 µl per reaction. TaqMan probes for human NPHS2 (podocin) (Applied Biosystems, catalog no. Hs00922492_m1) and rat NPHS2 (podocin) (Applied Biosystems, catalog no. Rn00709834_m1) were used. Standard curves were constructed for each assay using serially diluted cDNA standards. Podocin cDNA of known sequence and concentration was used as a standard for each assay so that the data could be calculated on a molar basis for each probe. Urine podocin mRNA was corrected per gram creatinine and expressed as urine podocin mRNA:creatinine. In 24-h urine podocyte mRNA analysis, urine podocyte mRNA measurements were also adjusted to 24 h and expressed as 24-h urine podocin mRNA.

### Kidney sample process for podometric analysis

The methods used were as previously described^17,29,30^ and were detailed as follows. For 3-week-old rats, the kidney was divided equally into two parts in cross section. For 2-month and 12-month rats, nearly 0.5 cm cylindrical kidney samples were collected near the renal hilum in cross sections. After embedding in paraffin, 3 µm thick kidney slices were cut and placed on slides. During immunofluorescence processing, these slides were deparaffinized in fresh xylene and rehydrated. After washing with PBS, the slides were permeabilized in Triton X-100. After that, antigen retrieval was performed by the high-pressure method. The slides were washed in PBS and then blocked with BSA for at least 2 h. The primary antibody (WT1 SC-7385, Santa Cruz) diluted 1:50 in 1% BSA was incubated overnight at 4 °C. On the second day, after a PBS wash step, the slides were incubated for 2 h at room temperature with Alexa Fluor 488 goat anti-mouse IgG (A11001; Invitrogen) diluted to 1:300 in 10% human serum diluted in PBS. After a washing step, the third antibody (A11055; Invitrogen) diluted at 1:300 in 10% human serum in PBS was incubated at room temperature for 2 h. Coverslips were mounted using SlowFade Gold anti-fade reagent with diamidino-2-phenylindole (S36939; Invitrogen). Then, glomerular images were collected under a fluorescence microscope (Leica DMI8). After immunofluorescence processing, random poles were chosen, and more than 30 consecutive glomeruli were captured by the “Z” method for further analysis. Then, the coverslip was removed, and the sections were rehydrated. The slides were then processed for immunohistochemical staining according to the manual instructions for the Vectastain Mouse IgG Kit (PK-6102; Vector). The slides were blocked in horse serum overnight at 4 °C. After a washing step, the slides were then incubated with primary antibody synaptopodin (SYNPO) at 1:500 (65294, Progen) for 2 h at room temperature. After incubation with the secondary antibody from the kit, the substrate diaminobenzidine (D4293 Sigma) was used to develop the brown peroxidase product. The slides were then washed and counterstained with hematoxylin, dehydrated, and mounted with resin. All the glomeruli were again imaged by microscopy (Olympus DP72).

This podometric analysis has been reported by many studies^17,29,30^. During this analysis, we first counted the apparent podocyte nuclear number (WT-1) in each glomerulus and measured the apparent glomerular area by tracing the glomerular outline by using Image-Pro Plus 6.0 software. After performing this measurement in more than 30 consecutive glomeruli in each sample, the mean apparent podocyte nuclear number and glomerular area will be determined. The section was cut at 3 µm thickness, and we assumed that the kidney sample in the section was a 3 µm high cylinder. Then, the volume of the section was calculated by multiplying 3 µm to the glomerular area measured by software. After dividing the nuclear number of podocytes to this volume and correction by the method established by Venkatareddy et al.^30^, the podocyte density was determined. The podocyte-positive area was estimated by the percentage of the SYNPO-positive area in the glomerular area.

### Kidney sample process for glomerulosclerotic index

Three-micrometer paraffin embedded slides were stained with PAS and then glomerulosclerotic index has been assessed at age of 12 months in both preterm and full term rats. Glomerulosclerosis was graded as follows: grade 0 indicating normal, grade 1 indicating sclerotic area up to 25%, grade 2 indicating sclerotic area 25–50%, grade 3 indicating sclerotic area 50–75%, and grade 4 indicating sclerotic area 75–100%. The glomerulosclerotic index score was evaluated as follows: score = (1 × n1) + (2 × n2) + (3 × n3) + (4 × n4)/n0 + n1 + n2 + n3 + n4. In this formula: nx is the number of glomeruli in each grade of glomerulosclerosis. 30 glomeruli per section were analyzed.

### Statistical analysis

GraphPad PRISM software, version 5.0 (GraphPad Software, Inc., USA), was used. T-tests were used to compare two groups. The chi-square test was used to compare differences in probability between two groups. The correlation between gestational age and UpodCR was analyzed by linear regression. When changes over multiple time points were examined, two-way ANOVA was employed and Bonferroni post hoc tests were used to compare groups at individual time points. A *P* value < 0.05 was considered statistically significant.

## Results

### Urine assay characteristics of preterm and full-term infants

Table 1 shows the demographics and clinical characteristics of full-term and preterm infants. Thirty-two full-term infants and 86 preterm infants were included in the present study.

**Table 1:** Characteristics of the full term and preterm infants.

Characteristics	Full term	Preterm	*P* value
Total no	32	86	NA
Male (%)	18 (56.3%)	49 (57.0%)	0.9435
Gestational weeks (Median) (range)	39 (37 + 2 to 41 + 1)	32 + 62 (24 + 3 to 36 + 5)	*< 0.05
Corrected gestational weeks when urine collected (median) (range)	39 + 5 (38 + 1 to 41 + 5)	39 + 2 (37 to 41 + 5)	0.1432
1 min apgar score (Mean ± SD)	9.81 ± 0.59	8.20 ± 1.35	*< 0.05
Cesarean section (No.) ( %)	9 (28.1%)	51 (59.3%)	*< 0.05
Birth weight (Mean ± SD) (gram)	3226 ± 329	1685 ± 569	*< 0.05
Weight at time point of urine collection (Mean ± SD) (gram)	3234 ± 324	2761 ± 357	* < 0.05

*P* value < 0.05 was considered statistically significant. Statistically significant differences are shown by asterisks.

NA: Not available.

### Rate of podocyte mRNA excretion in preterm versus full-term infants

**Figure 1:**
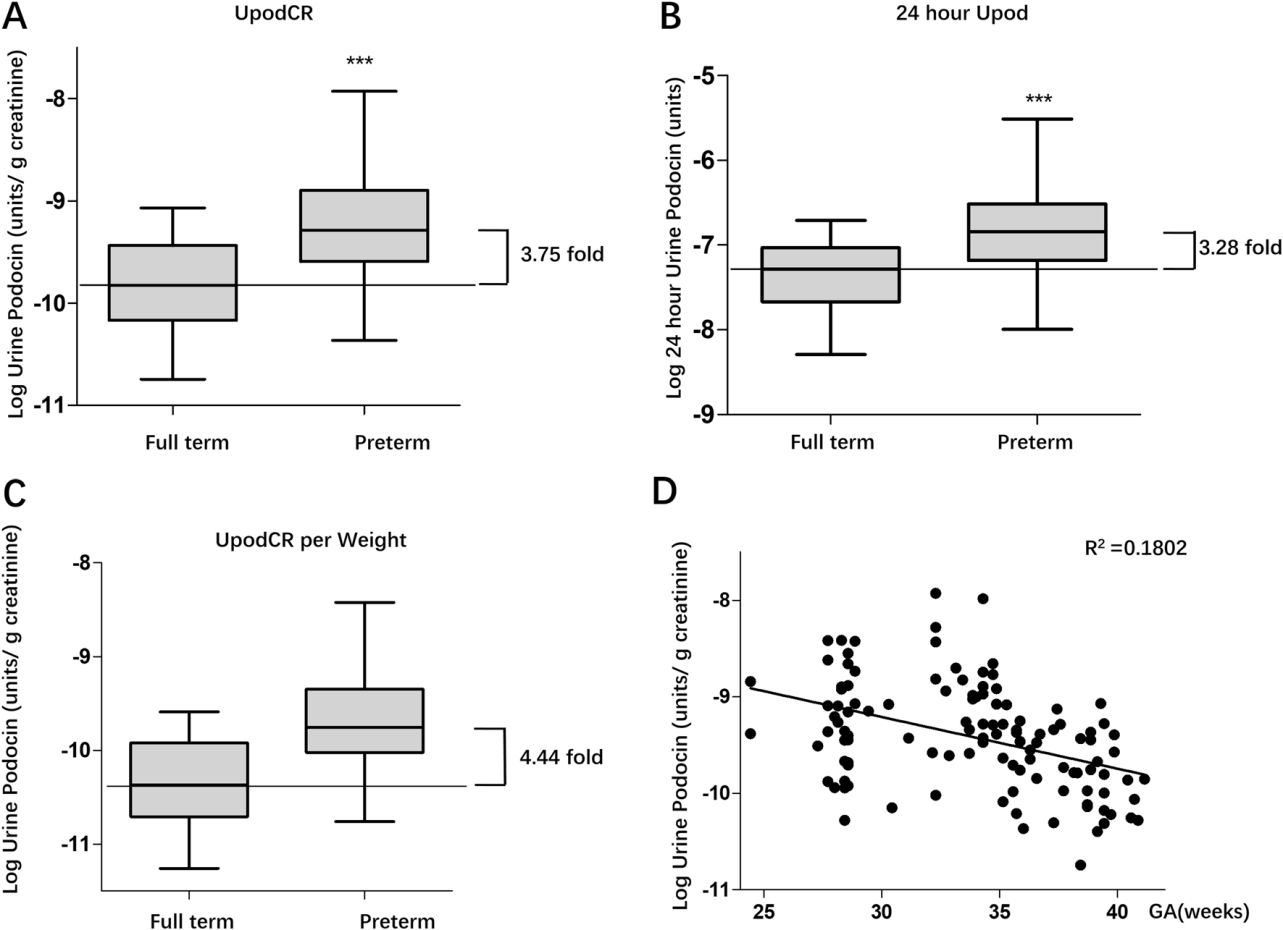
Increased urine podocyte mRNAs depletion rate in preterm infants. (**A**) Measurements of the rate of podocyte mRNAs depletion in the urine pellet were expressed as the urine podocin mRNA-to-creatinine ratio (analogous to the urine protein-to-creatinine ratio). Urine from full-term (n = 32) and preterm infants (n = 86) was compared. The preterm infants had a 3.75-fold increase in podocin mRNA in the urine pellet. (**B**) To exclude urine creatinine, which is related to the muscle mass effect on value of urine podocin mRNA-to-creatinine ratio, 24-h excretion of podocin mRNA were applied to examine the urine podocyte depletion rate. When comparing 24-h urine podocin mRNA between full-term (n = 32) and preterm infants (n = 86), preterm infant group presented a 3.28-fold increase. Statistically significant differences are shown by asterisks. (**C**) The urine podocin mRNA-to-creatinine ratio adjusted by weight is also presented, and the preterm infant group presented a 4.44-fold increase. Statistically significant differences are shown by asterisks. (**D**) Linear regression analysis was performed, and there was a negative correlation between gestational age at birth and urine podocyte excretion, with R^2^ = 0.1802. (T-tests were used to compare urine podocin mRNA levels shown in **A**–**C** between preterm and full term infants, ***P < 0.001).

The rate of podocyte mRNA excretion was estimated by detection of podocyte-specific podocin mRNA extracted from urine cell pellets. The final data are expressed as the ratio of urine podocin mRNA to creatinine (similar to the urine protein corrected by creatinine) to compensate for the variation in urine volume. As shown in Fig. 1A, the urine podocyte mRNA excretion rate was approximately 3.75-fold higher in preterm infants than in full-term infants (*P* < 0.001). Although age was matched between preterm and full-term infants, the weight of preterm infant was lighter than that of full-term infants at the time point of urine collection (Table 1). It is well known that urine creatinine is related to muscle mass and individuals with lighter weight usually present low urine creatinine. To exclude the effect of creatinine on urine podocin mRNA levels, we also expressed data as the 24-h urine podocin mRNA by application of 24-h urine volume multiplying podocin mRNA levels. As shown in Fig. 1B, urine podocyte specific mRNA was increased 3.28-fold in preterm infants compared with full-term infants (*P* < 0.001). In addition, urine podocin mRNA to creatinine was also adjusted by weight of infants and 4.44-fold increase of urine podocin mRNA level was found in preterm infants compared with full-term infants depletion (*P* < 0.001) (Fig. 1C). Moreover, there was negative correlation between gestational age at birth and urine podocyte mRNA excretion (Fig. 1D).

### Pattern of podocytes caused by preterm birth in a rat model

**Figure 2:**
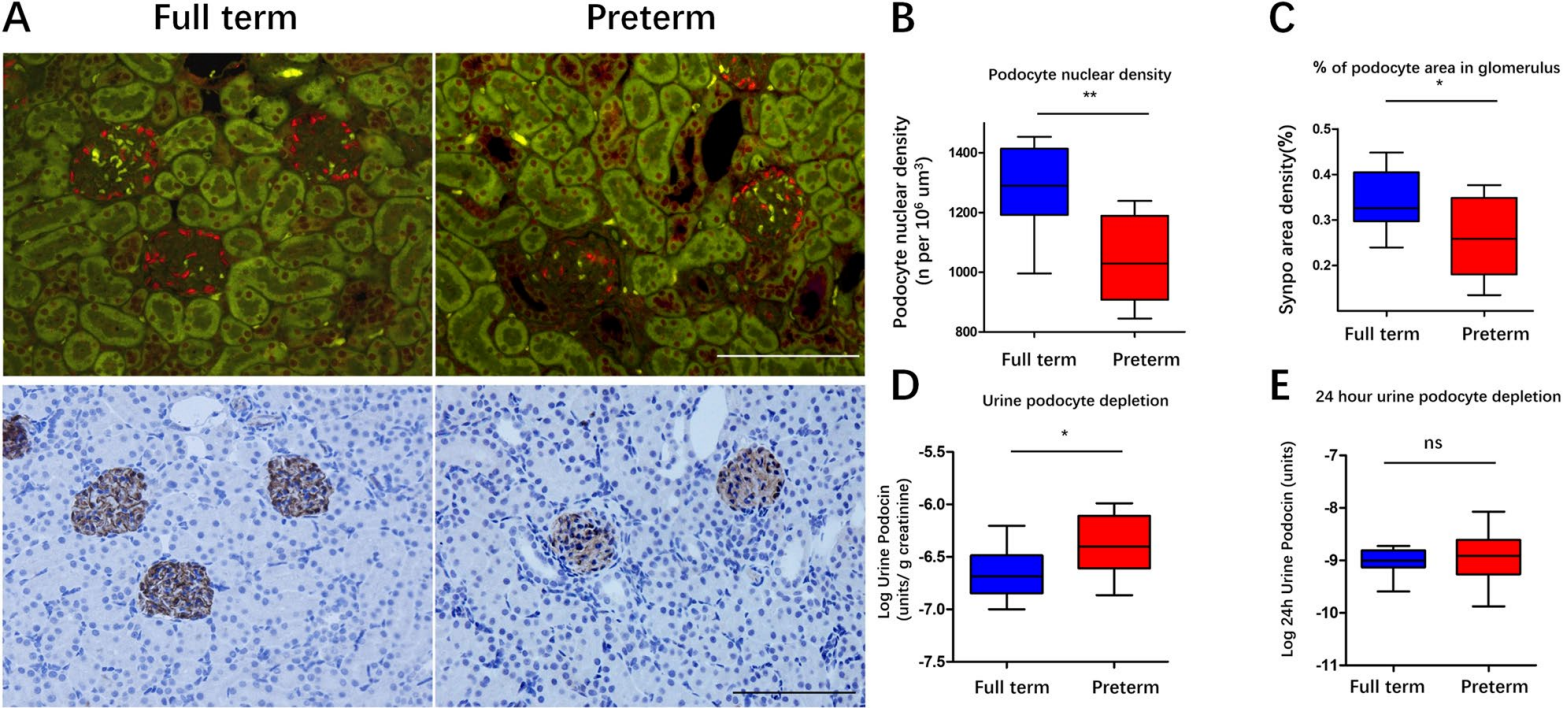
Effect of preterm birth on podocytes at postnatal week 3 in a rat model. (**A**) Podocyte density estimation by two independent methods. The upper panels show WT1-stained podocyte nuclei (red). The lower panels show SYNPO peroxidase-stained podocyte cell bodies in the same sections. Left panels illustrate full-term rat glomeruli. Right panels shown preterm rat glomeruli. Note that both podocyte nuclei and the podocyte area were decreased in preterm rats. Bar = 100 µm. (**B**, **C**) Biopsy podometric parameters of podocyte nuclear density and percent of podocyte aera in glomerulus showed that compared with the full-term rats (n = 9), the preterm rats (n = 8) have decreased podocyte numbers (*P* < 0.01) and podocyte area (*P* < 0.05). (**D**, **E**) Urine podometric parameters of the urine podocin mRNA-to-creatinine ratio (UpodCR) and the 24-h excretion of podocin mRNA (24 h Upod) are shown. The rate of podocyte mRNAs depletion in urine was increased in preterm rats compared to term rats when podocyte depletion was expressed as UpodCR (*P* < 0.05), while there was no significant difference when podocyte depletion was expressed as 24 h Upod. (T-tests were used to compare parameters between preterm and full-term rats, ***P < 0.001; **P < 0.01; *P < 0.05; ns: no statistically significant difference).

After establishment of the preterm rat model wherein pups were delivered 2 days early, podocytes were analyzed in the kidney at 3 weeks when the kidneys were completely differentiated with nephrogenesis undetected. As shown in Fig. 2A–C, compared with the full-term group, in the preterm group, podocyte nuclear density, which was expressed by the density of podocyte-specific nuclear protein WT-1, and the podocyte area density, which was expressed by the podocyte-specific protein SYNPO, were significantly decreased. Moreover, urine podocyte mRNA excretion in preterm rats was also detected and was proven to be higher than that in full-term rats (Fig. 2D).

### High risk of kidney disease caused by prematurity indicated by detection of podocytes mRNA in urine samples during long-term follow-up

**Figure 3:**
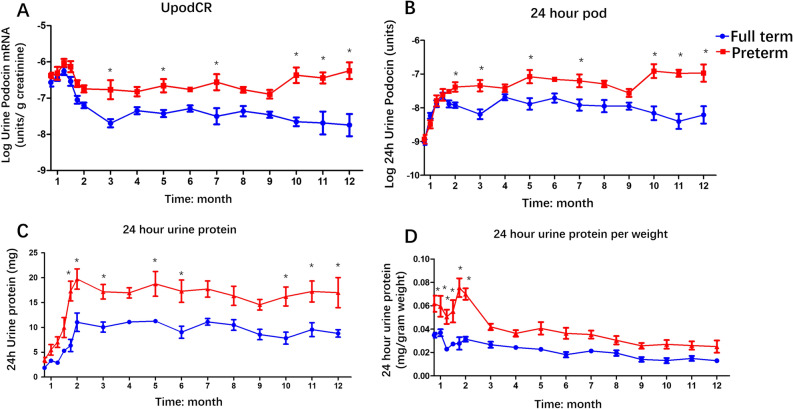
Time course of urine podocin mRNA depletion and urine protein in full-term rats and preterm rats (**A**, **B**). The rate of urine podocyte mRNAs depletion is expressed as the urine podocin mRNA-to-creatinine ratio (UpodCR) (**A**) and the 24-h excretion of podocin mRNA (24 h Upod) (**B**). At 3, 5, 7, 10, 11, 12 months of age in UpodCR and at 2, 2, 5, 7, 10, 11, 12 months of age in 24 h Upod, urine podocyte depletion was significantly increased in the preterm rats compared with the full-term rats at each time points. Statistically significant differences are shown by asterisks. (**C**, **D**) Proteinuria was expressed as 24-h urine protein (24 h UP) (C) and 24-h UP corrected by weight (D). Twenty-four-hour proteinuria was increased in the preterm rats over an all-time course from 3 weeks of age to 12 months of age compared with that in the full-term rats. At the ages of 1.75, 2, 3, 5, 6, 10, 11, and 12 month in 24 h UP and at the age of within 2 months in 24-h UP per weight, urine protein was significantly increased in preterm rats compared with full-term rats. (Two-way ANOVA was employed to examine changes over multiple time points and Bonferroni post-hoc tests were used to compare groups at individual time points. *P < 0.05).

The depletion of podocytes in the kidney and increase in the podocyte mRNA excretion rate in urine were initially represented in preterm rats at age of 3 weeks. To explore the effect of the initial pattern of change caused by preterm birth on long-term outcomes, urine samples were collected every week within 2 months and every month within 12 months. Both the urine podocyte mRNA excretion rate and urine protein were detected, as shown in Fig. 3, and consecutive high rates of podocyte mRNA excretion and proteinuria were detected in preterm rats. Regarding urine podocyte mRNA levels, significant differences were detected in total changes over time both in urine podocin mRNA to creatinine (Fig. 3A) (*P* < 0.001) and 24 h urine podocin mRNA (Fig. 3B) (*P* < 0.001) between the preterm group and the full term group. At individual time points, within the age of 2 months, there was no difference between preterm and full term rats in podocyte depletion. Differences were gradually examined at the age of 2–3 months between preterm and full term rats. Over the time, persistent podocyte mRNA excretion was detected from the age of 10–12 months (Fig. 3A,B). For urine protein, there were significant differences examined in total changes over time in 24-h urine protein (*P* < 0.001) and 24-h urine protein adjusted by weight (*P* < 0.001) between preterm and full term rats. At individual time points, there were differences detected at age of 7 weeks, and 2, 3, 5, 6, 10, 11, and 12 months in 24-h urine protein between preterm and full term rats (Fig. 3C) while there were differences only examined within the age of 2 months in 24-h urine protein adjusted by weight between preterm and full term rats (Fig. 3D). Moreover, there is significant time and different group interaction effect between preterm and term group in all four parameters (urine podocin mRNA to creatinine, 24-h urine podocin mRNA, 24-h urine protein, 24-h urine protein per weight) with *P* < 0.001, *P* < 0.001, *P* = 0.0132, *P* = 0.0076, respectively.

### High risk of kidney disease caused by prematurity indicated by detection of podocytes in kidney samples during long-term follow-up

**Figure 4:**
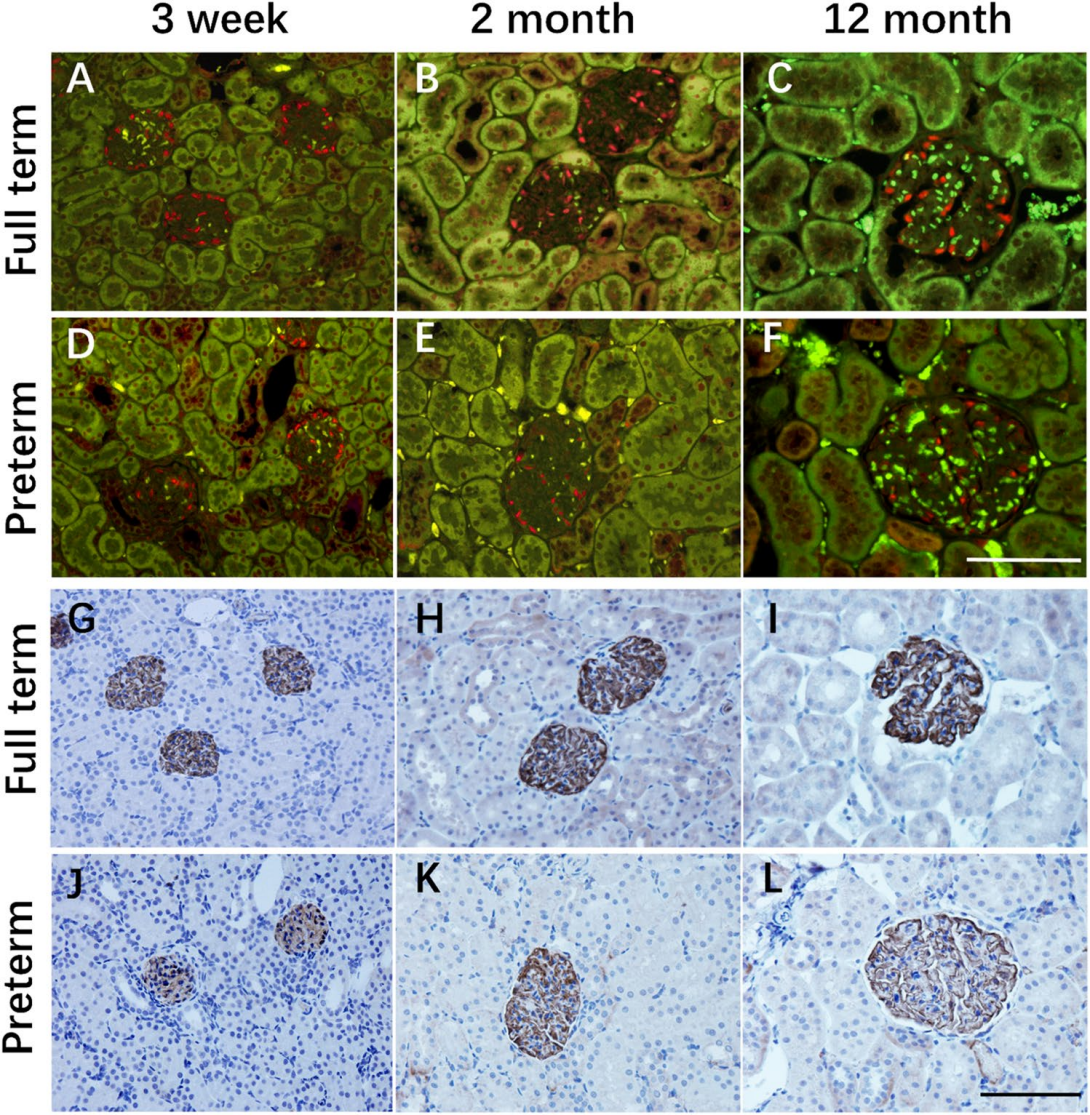
Time course of podocyte density presented by two independent methods in full-term and preterm rats. The upper two lines (**A**–**F**) show WT1-stained podocyte nuclei (red). The lower two lines (**G**–**L**) show SYNPO peroxidase-stained podocyte cell bodies in the same sections. The first and third lines (**A**–**C**, **G**–**I**) show glomeruli from full-term rats, while the second and fourth lines (**D**–**F**, **J**–**L**) show glomeruli from preterm rats. The left column illustrates glomeruli from 3 weeks of age (**A**, **D**, **G**, **J**). The middle column illustrates glomeruli from 2 months of age (**B**, **E**, **H**, **K**), and the right column shows glomeruli from 12 months of age (**C**, **F**, **I**, **L**). Both the podocyte nuclei density and podocyte cell bodies were reduced in the preterm rats. More detailed podometric parameters from these three time points are presented in Fig. 5. Bar = 100 µm.

**Figure 5:**
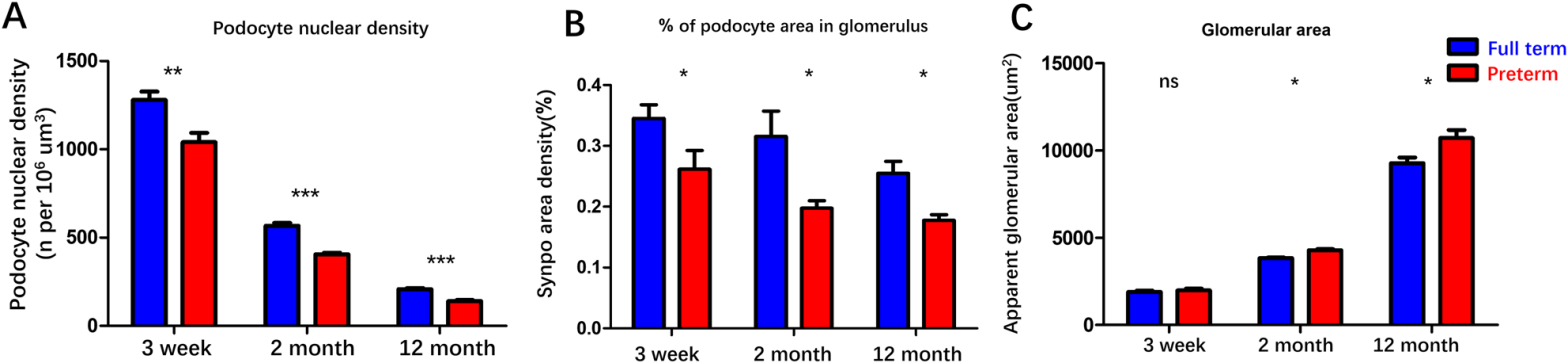
Podometric biopsy parameters in full-term and preterm rats at three time points. (**A**, **B**) Podocyte nuclear density and podocyte nuclear number per glomerulus in preterm rats were significantly decreased compared with those in full-term rats. In addition, podocyte nuclear density in preterm rats gradually decreased over time. Podocyte nuclear density in the preterm rats was decreased from 18.7 to 32.4% (mean value when compared with that of full-term rats). (**C**) The glomerular area was greater in the preterm rats compared to the term rats at 2 and 12 months. (***P < 0.001; **P < 0.01, *P < 0.05; ns: no statistically significant difference).

Kidneys were also collected from the ages of 2 months old and 12 months old, and podocyte counts were performed. As shown in Fig. 4, the WT-1 stained podocyte nuclear number per glomerulus and SYNPO stained podocyte area of preterm rats were lower than those of full-term rats (data presented in Fig. 5). At three time points, all significant differences were detected in podocyte nuclear density (Fig. 5A) and podocyte area density (Fig. 5B) between preterm and full term rats. When compared with the mean value of podocyte nuclear density between the two groups, podocyte density was found to decrease by 18.7%, 28.5%, 32.4% at the ages of 3 weeks, 2 months and 12 months, respectively, which showed the exacerbation of podocyte depletion over time (Fig. 5A). Moreover, glomerular enlargement was also found in the present study (Fig. 5C). There was no difference at the initial age of 3 weeks in glomerular area between preterm and full term rats. Then, significant differences were examined at the ages of 2 months and 12 months. The glomerular area was 1.11-fold larger and 1.16-fold larger in preterm rats than in full term rats at 2 months and 12 months, respectively (Fig. 5C). In addition, glomerulosclerotic index has been examined in full term rats and preterm rats at 12 months, there was no difference between two group (Mean ± SEM: 0.45 ± 0.056 vs 0.48 ± 0.047, *P* = 0.6408).

## Discussion

Prematurity is an important risk factor for long-term CKD^4–7^, but the pathogenesis involved is not very clear. The role of podocytes in the high risk of CKD caused by prematurity was explored in the present study. First, the major finding was a high rate of podocyte mRNA excretion in preterm infants compared with full-term infants (Fig. 1). The time point that we chose was corrected gestational week more than 36 weeks corresponding to the time of complete podocytes differentiation. In a preterm animal model, similar results were detected. At the age of 3 weeks, after nephrogenesis has ended, not only the urine podocyte mRNA excretion but also podocyte depletion from kidney samples were increased in preterm rats (Fig. 2), which indicated that podocyte and glomerular injury caused by prematurity.

Whether the initial injury caused by prematurity has long term effects, due to the lack of long-term follow-up data of preterm infants in children or adult stages, preterm rats were followed up until 12 months of age, corresponding to middle-aged cohorts in humans. Both the urine podocyte mRNA excretion and kidney podocyte depletion were examined along the time. As shown in Fig. 3, the overall trends of urine podocyte mRNA loss and urine protein excretion were different between preterm and full term rats over time. A higher rate of urine podoctye mRNA excretion and a higher value of urine protein were detected in preterm rats than in full term rats. At the individual time points, the post test results showed that at the early age of preterm birth (within 2 months), there were no differences between preterm and full term rats. However, a gradual difference was examined after the age of 2 months, and finally persistent podocyte mRNA excretion differences were examined at the age of 10 to 12 months (Fig. 3A,B). In addition, at kidney biopsy detection, gradually accelerated podocyte depletion was also indicated by podocyte nuclear density. These results all suggested that progressive podocyte abnormality was found over time. At age of 3 weeks, there was no significant difference in the glomerular area between the preterm and full-term rats. During that time, the glomerular area of the preterm rats was higher than that of the full-term rats at 2 months of age, which was approximately 1.11-fold larger than that of the full-term rats, and by 12 months of age, the increase in the glomerular volume of the preterm rats was 1.16-fold larger than that of the full-term rats (Fig. 5C). The increase in glomerular area suggested compensatory changes. According to the current podocyte depletion hypothesis^22,31–33^, accelerated podocyte loss and compensatory glomerular hypertrophy are the driving factor of future CKD, which suggests that prematurity is not only an important risk factor for CKD in the future but also confirms that accelerated podocyte loss plays an important role in the high risk of long-term CKD caused by prematurity. It is difficult to obtain human kidney biopsy specimens to detect which abnormalities in kidney as well as podocytes are caused by prematurity. Preterm rats were important animal model for exploring the details. Cary Stelloh et al. study reported low nephron number examined in preterm animal model^14^. In this study, the similar preterm rat model was applied and the biopsy podometrics was used to examine the abnormalities of podocytes. As shown in Fig. 2, both the podocytes nuclear density in glomeruli and the proportion of podocyte area in glomeruli were less than those in full-term rats at 3 weeks. At this time point, the podocytes have already been successfully differentiated. This finding suggested that the important effect on the kidney caused by prematurity was a reduction in podocytes in glomerulus. Podocytes are one of three layers of the glomerular filtration barrier. A low number of initially differentiated podocytes in glomeruli means a high workload of the remaining podocytes and vulnerability to kidney loss. The present study major focus on the impact of preterm birth on podocyte.

As previously shown in a glomerular disease animal model, loss of less than 20% of podocytes and hypertrophy of the remaining podocytes were initial changes during the progression of disease. When the loss reached 30%, the glomeruli became destabilized, which resulted in continuous podocyte loss. When the loss reached 70%, there was a decrease in serum creatinine and global sclerosis in pathological sections^21,29,34^. In the present study, the glomerulosclerosis index at the age of 12 months was also evaluated and there was no difference between the two groups. In a previous kidney disease rat model, the urine protein was 10–100-fold higher in the disease group than in the control group^21,34,35^. In the present study, the expression was only 2–3-fold higher in the preterm group than in the control group. For urine podocin mRNA, in kidney disease patients, urine podocin mRNA is more than ten times higher in patients than in controls^17,25,26^. In the present study, only an approximately threefold increase in podocyte podocin mRNA loss was found in preterm infants compared with full-term infants. Thus, prematurity is a mild risk factor rather than a pathogenic factor. Though the threefold increase in podocin mRNA is a minor change, it would be speculated an important risk factor for CKD associated with older age. In Naik et al. study, the same method was used in detection urine podocin mRNA in high-normal blood pressure individuals and sixfold increase were observed over the range of mean arterial pressure evaluated (70–111 mmHg)^36^. As researchers speculated based on previous study, this degree of increased podocin mRNA may also increase incidence of long-term progression associated with high normal mean arterial pressure^17,36–38^.

In the present study, during the follow-up of preterm rats, although the level of proteinuria in preterm rats was significantly higher than that in full-term rats, it had not yet reached the level of nephropathy (almost 10- to 100-fold higher in the disease group than in the control group)^21,35^. None of the preterm rats developed kidney diseases as adults. However, in the clinic, some preterm infants have developed kidney disease as adults, which suggests that preterm rat models are different from humans^4–7^. This may be caused by two main differences. One is gestational age. Previous studies suggest that the smaller the gestational age is, the higher the incidence of kidney disease in the future^6,7^. In premature infants, some extremely preterm infants have a very low gestational age of less than 28 weeks and have a higher incidence rate of long-term kidney diseases^7^. When these preterm infants were born, they experienced resuscitation and advanced life support, including supplementation with pulmonary surfactant and mechanical ventilation. In the preterm rat model of our study, a preterm rat model was established by delivery 2 days early, and the rats did not need advanced life support. In our pre-experiment, pups born 3 or 4 days early could hardly survive for more than 24 h because of their poor spontaneous breathing after birth. Therefore, we did not obtain a model in which the gestational age was too low. Second, in addition to congenital factors, the development of CKD is caused by many acquired factors, such as metabolic, immune, infectious, oxidant, and metabolic factors^16^. All of these factors target and accelerate podocyte loss during growth. In preterm infants, a higher occurrence of acute kidney injury, peripartum asphyxia, and drug exposure were confirmed in previous studies^39–41^. All of these factors can negatively affect the kidney as well as the remaining podocytes. In previous study, Kent et al. reported that preterm infants receiving indomethacin presented an increased number of podocytes in urine and higher excretion of albumin, which suggests glomerular injury^41^. Accumulated consumption of podocytes accelerates the development of kidney diseases. These complicated factors cannot be simulated in a preterm rat model.

Although the present study confirmed that accelerated podocyte mRNA loss in urine and podocyte loss in kidney play an important role in the development of long-term CKD, this may not be a single factor. Previous studies have confirmed that the detection of podocyte mRNA excretion is a more sensitive marker than proteinuria and creatinine. A significant increase in podocyte mRNA loss can be detected in kidney diseases negative for albuminuria or proteinuria^17,42,43^. However, in this study, at age of 3 weeks, a higher level of proteinuria in premature rats was detected, while the change in the podocyte mRNA excretion level was mild, even though there was no significant difference between preterm and full-term 24-h podocyte mRNA excretion. Previous studies have reported that preterm birth leads to a low number of nephrons in a preterm animal model^14^ and podocytes are part of the nephron, they do not comprise the whole nephron. The gestational age of preterm birth corresponds to the middle and late stages of kidney development. The development of the Loop of Henle (LOH) is one of the major developmental processes that occurs during these stages^44–46^. A low number of nephrons has been associated with an abnormal LOH. It is not clear whether extrauterine survival of preterm infants will have a great impact on LOH, and we look forward to future study of this issue.

There are several limitations in present study. First, there is a difference in kidney development between rats and humans. In humans, nephrogenesis is completed in utero approximately 36 gestational weeks before birth^12,27,28^, while nephrogenesis in rats is completed approximately between postnatal 10–15 days after birth^47,48^. In addition, infants in the preterm group were born at a very wide range of gestational ages, while rats in the preterm group were born at only one gestational age in the present study. Thus, the role of podocytes in long-term CKD concluded in rats could not completely indicate their role in humans. Second, in the present study, full-term control rats were obtained from vaginal spontaneous labor at 22.5 days of gestational age instead of cesarean delivery, which was performed in preterm rats. This is also limitation in our study. Third, in the present study, preterm birth presented increased podocyte loss, and the possible mechanisms of podocyte loss were not explored in the present study. In our latest study, we found that ribosome- and ribosome-related proteins that regulate mass protein synthesis have very important roles in the stages of mid- and late kidney development^49^. These stages corresponded to the stage of kidney development of preterm infants after birth. The abnormal regulation of ribosomes will likely occur in preterm infants, and then the reduction of total protein synthesis as well as protein in podocytes will occur. The detailed role of the mechanisms of podocyte loss caused by prematurity should be explored in future studies.

Increasing attention has been given to long-term disease in preterm infants. Our findings suggest that the acceleration of podocyte mRNA excretion in urine and podocyte loss in kidney are driving factor in increasing the risk of long-term CKD caused by prematurity.

## Data Availability

The data described in this manuscript are contained in published articles or available from the corresponding author upon reasonable request.
